# Handedness in twins: meta-analyses

**DOI:** 10.1186/s40359-021-00695-3

**Published:** 2022-01-15

**Authors:** Lena Sophie Pfeifer, Judith Schmitz, Marietta Papadatou-Pastou, Jutta Peterburs, Silvia Paracchini, Sebastian Ocklenburg

**Affiliations:** 1 Cognitive Psychology, Institute of Cognitive Neuroscience, Faculty of Psychology, Ruhr University Bochum, Universitätsstraße 150, 44780 Bochum, Germany; 2 School of Medicine, University of St Andrews, St Andrews, Scotland; 3 School of Education, Department of Primary Education, National and Kapodistrian University of Athens, Athens, Greece; 4 Biomedical Research Foundation of the Academy of Athens, Athens, Greece; 5 Institute of Systems Medicine and Department of Human Medicine, MSH Medical School Hamburg, Hamburg, Germany; 6 Department of Psychology, Medical School Hamburg, Hamburg, Germany

**Keywords:** Handedness, Twins, Meta-analysis, Laterality, Hemispheric asymmetry

## Abstract

**Background:**

In the general population, 10.6% of people favor their left hand over the right for motor tasks. Previous research suggests higher prevalence of atypical (left-, mixed-, or non-right-) handedness in (i) twins compared to singletons, and in (ii) monozygotic compared to dizygotic twins. Moreover, (iii) studies have shown a higher rate of handedness concordance in monozygotic compared to dizygotic twins, in line with genetic factors playing a role for handedness.

**Methods:**

By means of a systematic review, we identified 59 studies from previous literature and performed three sets of random effects meta-analyses on (i) twin-to-singleton Odds Ratios (21 studies, *n* = 189,422 individuals) and (ii) monozygotic-to-dizygotic twin Odds Ratios (48 studies, *n* = 63,295 individuals), both times for prevalence of left-, mixed-, and non-right-handedness. For monozygotic and dizygotic twin pairs we compared (iii) handedness concordance Odds Ratios (44 studies, *n* = 36,217 twin pairs). We also tested for potential effects of moderating variables, such as sex, age, the method used to assess handedness, and the twins’ zygosity.

**Results:**

We found (i) evidence for higher prevalence of left- (Odds Ratio = 1.40, 95% Confidence Interval = [1.26, 1.57]) and non-right- (Odds Ratio = 1.36, 95% Confidence Interval = [1.22, 1.52]), but not mixed-handedness (Odds Ratio = 1.08, 95% Confidence Interval = [0.52, 2.27]) among twins compared to singletons. We further showed a decrease in Odds Ratios in more recent studies (post-1975: Odds Ratio = 1.30, 95% Confidence Interval = [1.17, 1.45]) compared to earlier studies (pre-1975: Odds Ratio = 1.90, 95% Confidence Interval = [1.59–2.27]). While there was (ii) no difference between monozygotic and dizygotic twins regarding prevalence of left- (Odds Ratio = 0.98, 95% Confidence Interval = [0.89, 1.07]), mixed- (Odds Ratio = 0.96, 95% Confidence Interval = [0.46, 1.99]), or non-right-handedness (Odds Ratio = 1.01, 95% Confidence Interval = [0.91, 1.12]), we found that (iii) handedness concordance was elevated among monozygotic compared to dizygotic twin pairs (Odds Ratio = 1.11, 95% Confidence Interval = [1.06, 1.18]). By means of moderator analyses, we did not find evidence for effects of potentially confounding variables.

**Conclusion:**

We provide the largest and most comprehensive meta-analysis on handedness in twins. Although a raw, unadjusted analysis found a higher prevalence of left- and non-right-, but not mixed-handedness among twins compared to singletons, left-handedness was substantially more prevalent in earlier than in more recent studies. The single large, recent study which included birth weight, Apgar score and gestational age as covariates found no twin-singleton difference in handedness rate, but these covariates could not be included in the present meta-analysis. Together, the secular shift and the influence of covariates probably make it unsafe to conclude that twinning has a genuine relationship to handedness.

**Supplementary Information:**

The online version contains supplementary material available at 10.1186/s40359-021-00695-3.

## Introduction

Handedness is a form of human motor lateralization which has been studied extensively [1] as it is commonly understood as a proxy for functional brain lateralization [2]. Handedness shows a robust population-level asymmetry, with the great majority of people being right-handed and only 10.6% being left-handed as estimated by a recent meta-analysis [3].

However, left-handedness prevalence seems to vary in different populations. For example, it is well established that left-handedness occurs more often among males as compared to females [4]. Similarly, higher prevalence of atypical handedness has been reported in twins [5–9]. This finding was confirmed by Sicotte et al. [10] using meta-analysis. Without investigating moderators, the authors hypothesized that this effect could be mediated by pre- or perinatal circumstances which are more prevalent in twins or other form of multiples as compared to singletons [11–13]. For example, elevated proportions of left-handers were observed among singletons who experienced birth stress [14–16] and among children who were born preterm [17], by Caesarian section [18, 19], or struggled with breathing during birth [20]. Another aspect frequently associated with a tendency towards non-right-handedness is lower birth weight [21–23]. In a sample of Japanese and Dutch triplets, Heikkilä et al. [24] confirmed that left-handers displayed significantly lower birth weight than right-handers. In a recent large-scale study using the UK Biobank (*n* ~ 500,000), small but significant effects of birth year (increase in right-handedness of 0.7% per decade), birth weight (on average, right-handers are ~ 26 g heavier) as well as being part of a multiple birth (singletons = 9.5% left-handedness, multiples = 11.2% left-handedness, OR for right-handedness = 0.83) on handedness have been confirmed [25].

Sicotte et al. [10] also tested for differences in the prevalence of left-handedness between and monozygotic (MZ) and dizygotic (DZ) twins but found no effect.

As MZ twins share 100% of their DNA while DZ twins overlap on only 50% of genetic variants [2, 26], the twin model is often used to estimate heritability of one phenotypic trait [27]. A higher handedness concordance among MZ twins as compared to DZ twins [28–30] indicates a significant role of genetic factors in the ontogenesis of handedness. This was also confirmed by Sicotte et al. [10] (mean OR across studies = 1.37). Handedness heritability was estimated to be 0.24–0.26 in large samples of 21,127 twin pairs [31] or samples consisting of twins and their siblings adding up to 54,270 individuals [32]. Similarly, Somers et al. [33] estimated the heritability of left-handedness to be around 0.24 from a genetic linkage study in human pedigrees. In a large GWAS, Cuellar-Partida et al. [34] reported single nucleotide polymorphism (SNP) based heritability estimates of 5.9% for left-handedness and 12% for ambidexterity. This indicates that genetic factors account for up to one quarter of the variability of handedness.

Recently, several studies have been published on twin handedness. However, findings are not always in agreement, with different studies giving different estimates. For example, Zheng et al. [35] or Medland et al. [36] did not replicate a higher prevalence of atypical handedness in twins. Meta-analytic approaches can quantitively summarize the literature to provide an overall reliable estimate of handedness differences. Moreover, they can investigate possible small study bias in the literature and importantly allow for moderator analyses to investigate variables that could moderate the prevalence of handedness categories among twins [37]. Indeed, the vast field of handedness has recently seen an upsurge of meta-analyses that aim to summarize the literature and provide estimates of atypical handedness in various populations (e.g., individuals with autism [38], deaf individuals [39], intellectually disabled and intellectually gifted individuals [40], individuals with ADHD [41]).

Sicotte et al. [10] do report a meta-analysis of the handedness literature in twins. However, their meta-analysis was published more than 20 years ago, calling for an update as numerous new data sets have been published over the course of more than two decades. As an illustration, using the search term “handedness twins” on PubMed for publications that have been published after 1999 yields 120 hits. While not all of these studies might be eligible for meta-analysis, this number points towards a substantial increase in empirical studies over that period. Including this more recent data in meta-analysis is important, not only because it might result in more reliable estimates but also because antiquated efforts of forcing left-handers to use their right hand have largely been terminated [32, 42–44]. Moreover, the Sicotte et al. [10] analysis is limited by the fact that it only considered left- and right-handers. However, there is a certain proportion of people that cannot be classified in either of these categories. The definition of this mid-category is rather unsharp and its labelling varies from “mixed-handedness” over “both-handedness” to “ambidexterity”. As emphasized by Papadatou-Pastou et al. [3], even if these terms are often used interchangeably, “ambidextrous” refers to individuals being equally skilled with both hands while “mixed-handed” refers to individuals preferring to use different hands for different tasks. When handedness is determined as self-report of writing hand, it is thus by definition only possible to account for ambidexterity, but not mixed-handedness. In contrast, self-report questionnaires like the Edinburgh Handedness Inventory [45] assess the preferred hand for several manual activities, which therefore captures ambidextrous as well as mixed-handed individuals in the mid-category. Consequently, the meta-analysis by Papadatou-Pastou et al. [3] confirmed that the method to determine handedness affects precise point estimates of atypical handedness prevalence. The authors further found that the prevalence of this mid-category is 9.3%, suggesting that a strong lateralization towards the right side is the common rule, whereas non-right-handedness (including left-, mixed-handedness and ambidexterity) is generally referred to as “atypical” handedness [3]. All in all, newly gathered insights may be capable of challenging the interpretations made by Sicotte et al. [10], and recent accumulations in overall data might even allow for divergent results.

Thus, the major goal of the present meta-analysis is to update the state of the art concerning the questions of whether atypical handedness occurs more often in twins than in singletons. Three sets of meta-analyses were conducted. Firstly, we compared the prevalence of atypical handedness in twins and singletons. Secondly, we examined whether atypical handedness occurs more often in MZ compared to DZ twins. Thirdly, we analyzed data on handedness in twins in a pairwise manner to test whether MZ and DZ twin pairs differ in their prevalence of handedness concordance. Beyond those three sets of meta-analyses, we performed various moderator analyses to elucidate whether additional factors such as inclusion in the Sicotte et al. [10] meta-analysis, method of determining zygosity, sex, age, year of publication, measurement of handedness, handedness classification, nature of the singleton group, and purpose of the study moderated potential differences in atypical handedness prevalence in twins and singletons.

## Materials and methods

### Selection of studies for the meta-analyses

The study selection by means of a systematic review as well as the conduction of all meta-analyses in this study followed the official PRISMA guidelines [46, 47]. As it is the aim of the PRISMA guidelines to increase the traceability of reviews and meta-analyses, it includes a concrete 27-item checklist which we applied for the selection and inclusion of studies in our meta-analyses (Additional file 2). Risk-of bias (also called critical appraisal) analysis was not deemed necessary for our included studies, because they were not assessing an intervention (therefore elements like blinding participants and randomization were not relevant) or an experimental manipulation (therefore elements like blinding of the experimenters were not relevant). Moreover, we only included published studies that may be assumed to have sufficient quality as a result of peer-review processes. However, we did check for various methodological qualities of our included studies, such as measurement of handedness, purpose of the study or way to determine zygosity in the context of several moderator analyses (see below).

**Fig. 1:**
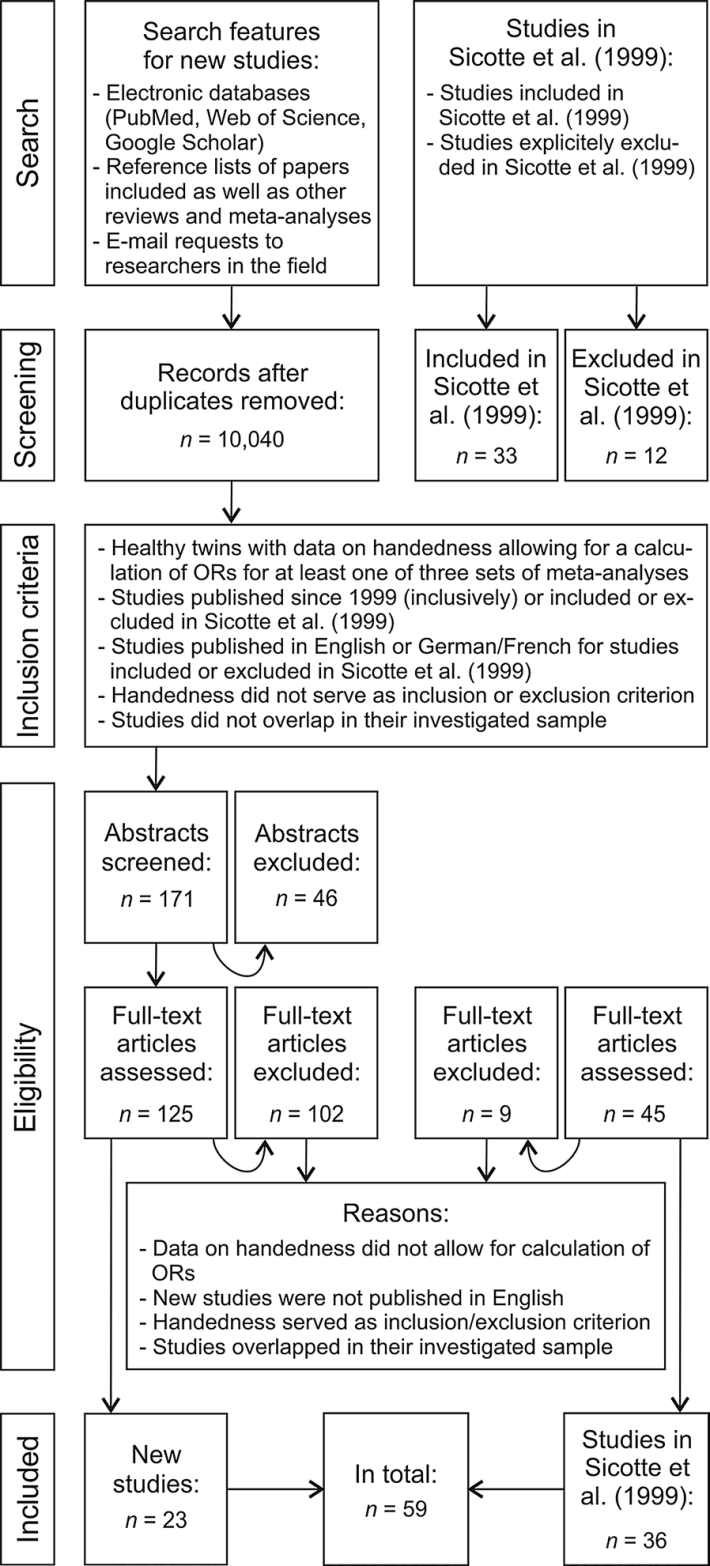
Flow diagram depicting criteria from the PRISMA guidelines for systematic reviews and meta-analyses as well as inclusion and exclusion criteria which were applied in the course of search and inclusion of studies for these meta-analyses. Additional file 1: Table S1 contains a comprehensive list of all studies included in our meta-analyses

The purpose of this study was to review and reanalyze the meta-analysis by Sicotte et al. [10] as well as to seek and aggregate new data on handedness in twins to update the state of the art. Therefore, we opted to combine the data of studies included in the meta-analysis by Sicotte et al. [10] with new data from recent studies which were identified in the course of an extensive literature search. If studies were not accessible online, local databases were searched for the respective articles or corresponding authors were contacted via e-mail requests when possible. Data collection as well as extraction was conducted by LP and concluded in September 2020. Details of this process are shown in Fig. 1. Data collection and extraction were evaluated by SO and discrepancies were resolved by discussion.

### Inclusion and exclusion criteria

The following inclusion and exclusion criteria were applied:

1. Data: Studies needed to provide data on handedness in twins. For inclusion, studies either needed to allow (a) for a calculation of Odds Ratios (ORs) for a comparison of handedness between twins and singletons, (b) for a calculation of ORs for a comparison of handedness between MZ and DZ twins, or (c) for a calculation of ORs for a comparison of handedness concordance between MZ and DZ twins. In cases where studies reported arithmetic data in a way that did not allow for the calculation of ORs used in the meta-analyses (e.g., laterality indices, averages, quotients), we contacted the authors to ask for more specific information on the distribution of handedness groups across the sample. Studies were excluded if the authors did not provide that additional information.
2. Language: Studies had to be written in English to be included in our meta-analyses. Exceptions were made for the studies published in German or French which were included in the analysis by Sicotte et al. [10]. Concerning the German studies, we extracted the data ourselves, whereas for studies written in French we relied on the data extraction performed by Sicotte et al. [10].
3. Handedness: As it was our goal to investigate the prevalence of atypical handedness in twins, we excluded studies in which handedness was defined as an inclusion or exclusion criterion (e.g., left-handedness as exclusion criterion, participants matched for or selected on the basis of handedness or concordance/discordance for handedness).
4. Participants: As atypical handedness patterns are associated with several psychiatric [48–50] and neurodevelopmental [38] conditions, studies needed to provide data on handedness for healthy twins. In cases where mixed samples were examined [51–58], we only extracted data on handedness for twin pairs concordantly healthy who served as control twins in these studies. Therefore, the report of handedness data had to be precise enough to clearly distinguish between healthy control twins and affected twins (in cases where twins were discordant for conditions, we opted to also exclude the healthy co-twin). Likewise, when studies compared the handedness of twins and other multiples with sib-pairs or singletons, handedness had to be reported separately for those groups. For studies which did not report the data precisely enough for the mentioned groups, we contacted the authors to ask for additional information. Studies were excluded if the authors did not provide this information. In total, we included 59 studies (including 32 studies already included in Sicotte et al. [10]) in our meta-analyses (Fig. 1). A comprehensive list of all included studies is presented in Additional files 1, 2. Studies included in the meta-analyses are marked with asterisks in the reference list. 

### Studies included in the meta-analysis by Sicotte et al. (1999)

We aimed to include the studies analyzed by Sicotte et al. [10] but screened them against our inclusion and exclusion criteria (see above) as those slightly deviated from the ones applied by Sicotte et al. [10]. In detail, these authors included all studies containing at least ten twin pairs and providing data on two or more groups of individuals. As a result, we excluded one study [7] because it seemed to contain other forms of multiples apart from twins (e.g., triplets) and reported data on handedness in a combined manner for them. Furthermore, we checked twelve studies which were explicitly reported to have been excluded in the meta-analysis by Sicotte et al. [10]. We opted to include four of these because they fulfilled our inclusion criteria. In detail, Sicotte et al. [10] excluded these studies due to incorrect references [59] or the lack of pair-wise data [60, 61]. In contrast, we were able to use these studies for at least one of our comparisons. Moreover, Sicotte et al. [10] excluded two studies [60, 62] as the exact number of twins was not stated. As we were able to calculate the number, we could include both studies. Overall, we analyzed 32 studies included in the meta-analysis by Sicotte et al. [10] and four studies explicitly excluded by Sicotte et al. [10] providing data on handedness in twins covering publications from 1924 to 1996.

### New studies

New data were collected by means of literature search for all studies that reported handedness for twins (regardless of whether it was the original purpose of the study to examine handedness or not) and that had been published since 1999 (inclusively). Thereby, we tried to ensure including all studies not covered by Sicotte et al. [10] as they reported having conducted their search for studies from 1966 to “present” so that we assumed their latest results to cover the years 1998/1999. In detail, the electronic databases PubMed (https://www.ncbi.nlm.nih.gov/pubmed/), Web of Science (https://www.webofknowledge.com), and Google Scholar (https://scholar.google.de/) were searched for the terms “handedness” AND “twins”, “hand preference” AND “twins”, “hand skill” AND “twins” and “twins” AND “pegboard”. By means of these search terms, we further extended the work by Sicotte et al. [10] who restricted their literature review to the keywords “twins” and “handedness”. Reference lists of included papers as well as other reviews and meta-analyses were further used as source to identify further studies [2, 31, 36, 63, 64]. This is in line with the search by Sicotte et al. [10] who similarly included studies that were identified in prior reviews.

### Overlapping datasets

In cases where the same data were used by more than one study, the dataset was included in our analyses only once. We checked overlapping studies separately for the three sets of meta-analyses we performed, as it was conceivable that the same dataset was depicted in different ways by different studies so that one publication might have allowed extraction of the data for our first set of meta-analyses while another publication on the same sample might have allowed extraction of the data for the second set of meta-analyses.

First, the twins included in Segal [65] and Gopalakrishman [66] seemed to overlap with the twins investigated by Sicotte et al. [10], so we could not include those new studies.

For new studies overlapping in their investigated datasets, we opted to include the oldest study, with the exception when a more recent study included a larger dataset. Specifically, Hulshoff Pol et al. [67] seemed to overlap with Bootsman [68] for the Netherlands Twin Registry. As Hulshoff Pol et al. [67] was older and included more data, we opted to include this study and to exclude Bootsman [68].

Similarly, Vuoksimaa et al. [69] seemed to overlap with several studies [70–76] for the Older Finnish Twin Cohort of same-sex twin pairs born in Finland before 1958. As Vuoksimaa et al. [69] provided the most data on this sample, we chose to include this study and to exclude all others. Heikkilä et al. [77] also seemed to report data on this sample by means of the FinnTwin12 cohort. However, this study also included the FinnTwinn16 cohort, so we extracted data only for this dataset out of Heikkilä et al. [77]. Moreover, Heikkilä [78] overlapped with Heikkilä et al. [77]. The latter was a doctoral dissertation in which this study as well as two others (which we assessed and excluded in the process of our data collection for this meta-analysis) were included. Therefore, we opted to include Heikkilä et al. [77] and to exclude Heikkilä [78].

Moreover, several studies overlapped for Australian twin samples. Medland et al. [79] included two samples of which only the second one allowed for the second and the third set of meta-analyses. However, this sample was based on the Brisbane Adolescent Twin Study which was also described in Medland et al. [36]. As Medland et al. [36] was older and provided far more data, we opted to include this study to account for Australian twins. As a result, we also had to exclude Kanchibhotla et al. [80] as this study was based on the Australian Twin Registry which was already covered by Medland et al. [36] as well. As Dooland et al. [81] reported dental schools in Adelaide and Melbourne as their primary source of recruitment, this study did not overlap with Medland et al. [36] and was therefore included. Finally, data reported in Medland et al. [36] were extracted from Medland et al. [31] as they were reported in more detail in that article. Similarly, pairwise data had not been reported by Basso et al. [82] and were extracted from Medland et al. [31] who reported the pairwise data after having contacted the original authors.

### Data extraction

We relied on the data extraction performed by Sicotte et al. [10] for five studies as they were either written in French [28, 83, 84], we had no access to it [85], or Sicotte et al. [10] reported far more data than we could find, assuming that they had received additional material by the original study authors [86].

For all other studies reported by Sicotte et al. [10], we extracted the data from the original papers. In cases where handedness data for individuals and pairs were conflicting (e.g., when not all individuals originated from complete pairs), we opted for the individual data. Nevertheless, in the context of our third set of meta-analyses, we acknowledged pairwise data but concentrated on handedness concordance or discordance of pairs not taking into account information on the specific handedness direction (e.g., for concordant pairs, we did not distinguish between R-R- (both twins right-handed), L-L- (both twins left-handed), or A-A- (both twins mixed-handed/ambidextrous) pairs). Likewise, data extraction for our meta-analyses partly resulted in some deviations from the data reported by Sicotte et al. [10]. For instance, we extracted data on handedness categories as detailed as possible using mixed-handedness as its own handedness category. Sicotte et al. [10], in contrast, subsumed individuals reported to be ambidextrous in the original studies under left-handers, thus reducing detail by only distinguishing between right- and left-handedness.

### Statistical analysis

All meta-analyses were performed in R using the metafor package [87]. To address our research questions, we performed the following three sets of meta-analyses:

- *Meta-analysis set 1*: The first set of meta-analyses addressed the question of whether twins and singletons differ in their prevalence of atypical handedness (left-handedness, mixed-handedness, or non-right-handedness). This analysis was run on all studies that provided separate handedness data for twins and singletons (21 studies). Odds Ratios (ORs) were calculated for twins vs. singletons for left-, mixed-, and non-right-handedness. An OR of 1 is indicative of no group difference, while ORs > 1 suggest a higher prevalence of atypical handedness in twins compared to singletons and ORs < 1 suggest a higher prevalence of atypical handedness in singletons compared to twins. Random effects models were run on the ORs for left-, mixed-, and non-right-handedness, followed by a moderator variable analysis (see below).

**Fig. 2:**
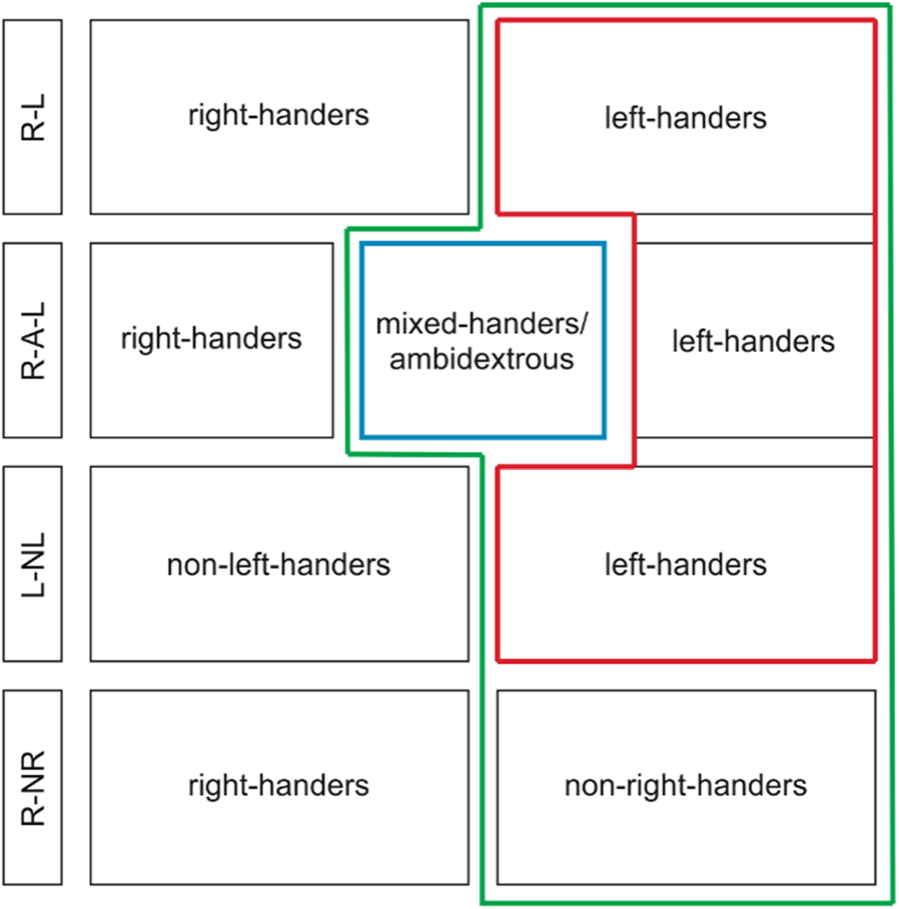
Visualisation of the atypical handedness groups per classification. The red box represents groups included in the left-handedness comparison, the blue box represents the group included in the mixed-handedness comparison, and the green box represents groups included in the non-right-handedness comparison

The atypical handedness groups correspond to the following:

1. The left-handedness group included left-handers from the “right vs. left” (R-L), “right vs. ambidextrous/mixed-handed vs. left” (R-A-L), and “left vs. non-left” (L-NL) classifications (red box in Fig. 2).
2. The mixed-handedness group included mixed-handers in the R-A-L classification (blue box in Fig. 2). The nature of this group depends on the instrument used to assess handedness. For example, studies using a writing hand criterion (e.g. Vuoksimaa et al. [69]) identify ambidextrous individuals (who use both hands for writing), as their middle category, while studies using several hand preference items (e.g. Shimizu et al. [88]), also identify mixed-handed individuals (who use the left hand for some tasks and the right hand for other tasks). Here, we generally refer to the mid-category as it was defined by the original studies (individuals that were not assigned to the group of right-handers or left-handers) when referring to ‘mixed-handedness’. Therefore, the mixed-handed group consists of both mixed-handers and ambidextrous individuals.
3. The non-right-handedness group included left-handers (R-L and R-A-L), mixed-handers (R-A-L), and non-right-handers (“right vs. non-right”, R-NR) (green box in Fig. 2).

- *Meta-analysis set 2*: The second set of meta-analyses addressed the question of whether MZ and DZ twins differ in their prevalence of left-, mixed-, or non-right-handedness. This analysis was run on all studies that provided separate handedness data for MZ and DZ twins (48 studies). ORs were calculated for MZ vs. DZ twins for left-, mixed-, and non-right-handedness. ORs > 1 suggest higher prevalence of atypical handedness in MZ twins compared to DZ twins, and ORs < 1 suggest higher prevalence of atypical handedness in DZ twins compared to MZ twins. We ran random effects models on the ORs for left-, mixed-, and non-right-handedness, followed by a moderator variable analysis (see below).
- *Meta-analysis set 3*: The third set of meta-analyses addressed the question of whether MZ and DZ twin pairs differ in the prevalence of pairwise handedness concordance. This analysis was run on all studies that provided pairwise handedness data for MZ and DZ twins (44 studies). An OR was calculated for handedness concordance in MZ vs. DZ twins. An OR > 1 suggests higher concordance in MZ twins compared to DZ twins, and an OR < 1 suggests higher concordance in DZ twins compared to MZ twins. We ran a random effects model on concordance OR and subsequently ran a moderator variable analysis (see below).

### Study heterogeneity and small study bias

For each meta-analysis, we tested for homogeneity using the *I*^*2*^ index reflecting the variance explained by heterogeneity across studies. The *I*^*2*^ index is assumed to be low, moderate, and high, when it takes values close to 25%, 50%, and 75% respectively [89]. The Tau^2^ index was used to specify variance between studies. We visually inspected the funnel plot created using the funnel() function to identify small study bias. Funnel plot asymmetry was also assessed using Egger’s regression test (regtest() function). Finally, the trim and fill method (trimfill() function) [90] was used to impute data points in order to make the funnel plot symmetrical.

### Moderator analyses and variables

1. Sicotte et al. (1999) meta-analysis: In order to compare our results with those obtained by Sicotte et al. [10], we first tested for an effect of inclusion in the Sicotte et al. (1999) meta-analysis (included in Sicotte et al. [10], excluded from Sicotte et al. [10], new studies) on ORs. This analysis was run for all three sets of meta-analyses (1, 2, and 3).
2. Year of publication: As it has been shown that early studies bias the distribution of handedness categories [3], we tested for any moderating effects of the year of publication of the original study on ORs in the twins vs. singletons meta-analysis (meta-analysis set 1).
3. Ancestry: As handedness is believed to be partially genetically determined, we investigated moderating effects of ancestry in terms of the genetical origin of the participants of the original studies. In this context we distinguished between (a) Europe/USA/Australia and (b) East Asia. This analysis was run for the twins vs. singletons meta-analysis (meta-analysis set 1).
4. Purpose of the study: We investigated if there was any moderating effect of whether (a) it was the original purpose of the study to examine handedness in twins, or (b) whether the study only reported data on handedness as a descriptive variable independent of the research question of the study. This analysis was run for the twins vs. singletons meta-analysis (meta-analysis set 1).
5. Sex ratio: As confirmed by a meta-analysis by Papadatou-Pastou et al. [4], males display higher rates of left-handedness than females. When numbers for males and females were reported, we investigated whether the male:female sex ratio had any moderating effect on ORs. This analysis was run for the twins vs. singletons meta-analysis (meta-analysis set 1). We did not perform analyses separately for males and females as data on handedness were rarely broken down by sex separately for twins and singletons.
6. Mean age of the participants: We investigated whether the mean age of the participants had any moderating effect on the ORs for atypical handedness between twins and singletons (meta-analysis set 1).
7. Type of singleton group: Since handedness is believed to be partially genetically determined, we investigated whether there was any moderating effect on the ORs for atypical handedness between twins and singletons (meta-analysis set 1) depending on (a) whether twins and singletons were genetically related (e.g., singletons were siblings of twins) or (b) not.
8. Handedness classification: We investigated whether the handedness classification had any moderating effect on the ORs for atypical handedness between twins and singletons (meta-analysis set 1). Here, we distinguished between the classification schemes of (a) “right vs. ambidextrous/mixed-handed vs. left” (R-A-L) and (b) “right vs. left” (R-L).
9. Method of handedness assessment: As it was shown that handedness assessment affects handedness outcomes [3], we investigated whether the assessment method had any moderating effect on the ORs for atypical handedness between twins and singletons (meta-analysis set 1). Those methods varied between (a) preference obtained from performance inventories in which the individuals’ handedness was determined on the basis of more than one item and (b) self-reports/writing hand.
10. Method of determining zygosity: We investigated whether the method of determining zygosity had any moderating effect on the ORs in the MZ vs. DZ (meta-analysis set 2) and in the concordance analysis (meta-analysis set 3). In this context, we distinguished between (a) serological and genetic methods and (b) questionnaires and observational methods.

For most studies, not all the variables of interest were reported. Therefore, the number of studies included in each of the three sets of meta-analyses as well as in the moderator analyses varied. Hand skill was very rarely reported. In cases where hand skill and hand preference were reported [91], we opted to extract data for hand preference. When studies used handedness inventories containing several items but reported handedness prevalence for every item separately (e.g., Zheng et al. [35], we extracted data for writing hand, as this is the most commonly used measure for handedness [3].

Moderator analyses were conducted for the non-right-handedness and the left-handedness classification schemes. The mixed-handedness classification scheme included only *n* = 5 and *n* = 10 studies in meta-analysis set 1 and 2, respectively, therefore not allowing for this kind of analysis.

## Results

### Meta-analysis set 1: prevalence of atypical handedness in twins vs. singletons

The aim of the first set of meta-analyses was to reveal whether there was higher prevalence of atypical handedness (left-handedness, mixed-handedness, or non-right-handedness) in twins compared to singletons. Overall, 21 studies (13 included by Sicotte et al. [10], one excluded by Sicotte et al. [10], seven new studies) allowed for the calculation of ORs for twins vs. singletons, including *n* = 139,242 singletons, and *n* = 50,180 twin individuals, resulting in a total sample size of *n* = 189,422 individuals.

**Fig. 3:**
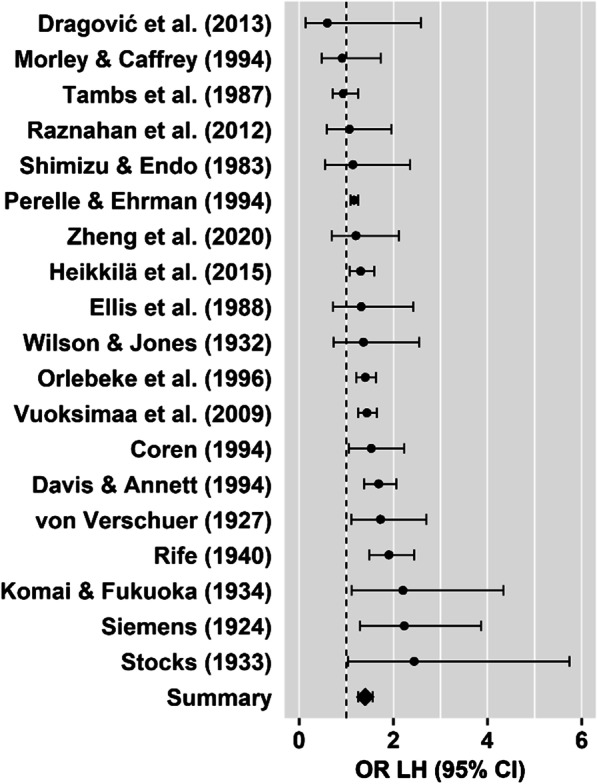
Forest plot for the twin-to-singleton left-handedness meta-analysis. The dots represent ORs for each study and horizontal lines represent the 95% confidence intervals. The summary OR suggests higher prevalence of left-handedness in twins compared to singletons

**Fig. 4:**
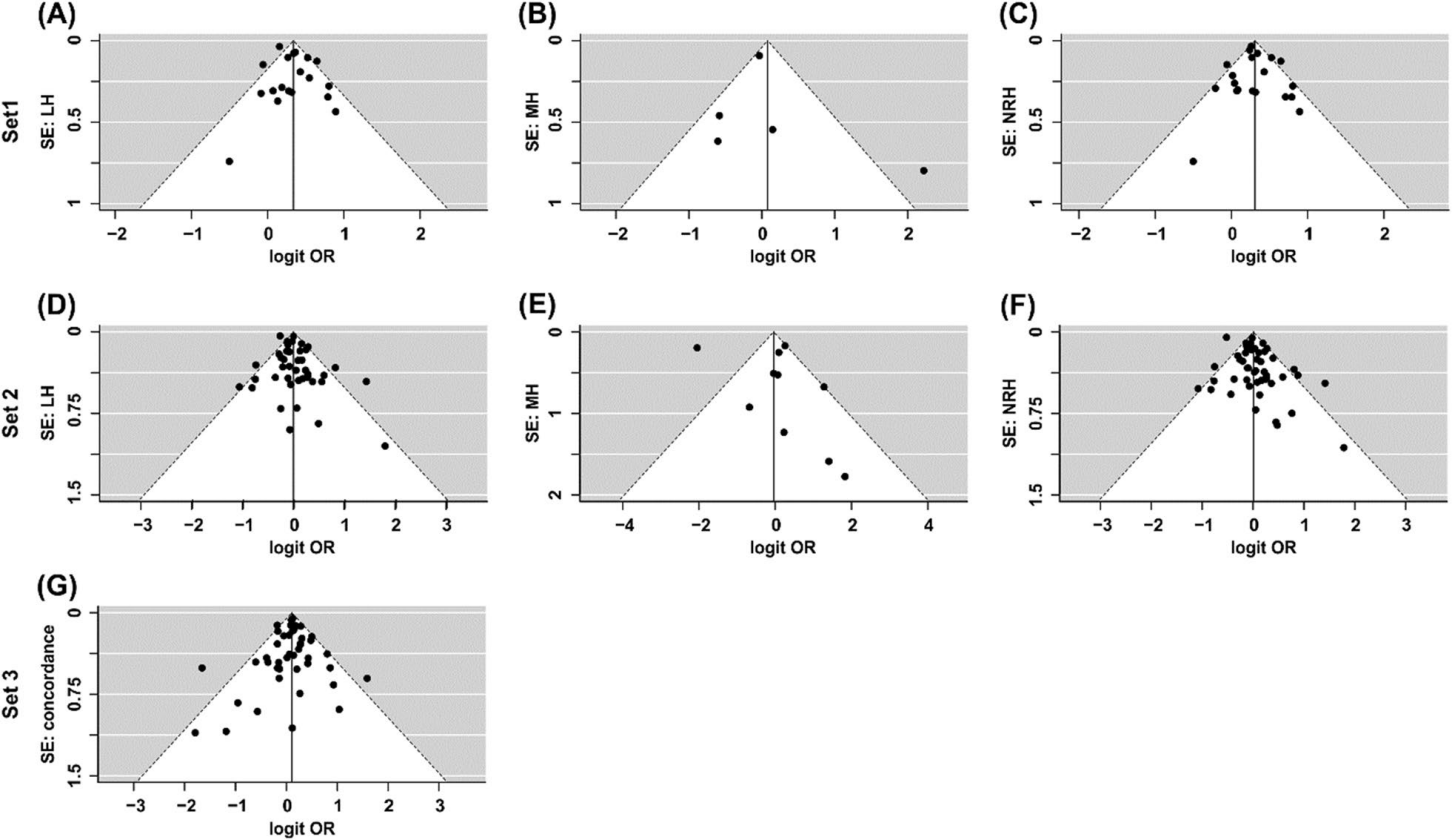
Funnel plot of standard errors on logit prevalence. Funnel plots **a** “LH” (left-handedness), **b** “MH” (mixed-handedness), and **c** “NRH”(non-right-handedness) refer to meta-analysis set 1 (twins vs. singletons), and by means of a visual inspection no asymmetries could be identified. Funnel plots **d** “LH”, **e** “MH”, and **f** “NRH” refer to meta-analysis set 2 (DZ vs. MZ), and according to visual inspection we detected no asymmetries. Funnel plot **g** “concordance” refers to meta-analysis set 3 (concordance), and a visual inspection did not reveal any asymmetry

*Left-handedness:* The twin-to-singleton left-handedness OR provided evidence for a higher prevalence of left-handedness in twins (Table 1, Fig. 3) with moderate to high heterogeneity among the studies (*p* < 0.001). Neither Egger’s regression test for funnel plot asymmetry (*z* = 0.11, *p* = 0.909), visual inspection of the funnel plot (Fig. 4a), nor the trim and fill test (0 studies to impute, SE = 2.67) revealed evidence for small study bias.

**Table 1:** Results of meta-analysis set 1

	Left-handedness	Mixed-handedness	Non-right-handedness
Studies (*k*)	19	5	20
	13 included in [10]	2 included in [10]	13 included in [10]
	1 excluded from [10]	3 new studies	1 excluded from [10]
	5 new studies		6 new studies
Individuals in total (*n*)	189,422		
Individuals per comparison (*n*)	188,922	39,123	189,274
Twins (*n*)	49,881	26,625	50,066
Singletons (*n*)	139,041	12,498	139,208
Prevalence in twins	9.13% (*n* = 4552)	3.39% (*n* = 903)	11.11% (*n* = 5564)
Prevalence in singletons	6.97% (*n* = 9692)	2.67% (*n* = 334)	7.23% (*n* = 10,069)
OR [95% CI]	1.40 [1.26, 1.57]	1.08 [0.52, 2.27]	1.36 [1.22, 1.52]
z	5.98***	0.21	5.65***
Heterogeneity among studies	Q(18) = 45.42***	Q(4) = 10.39*	Q(19) = 37.94**
	I^2^ = 60.39%	I^2^ = 72.68%	I^2^ = 61.06%
	Tau^2^ = 0.02	Tau^2^ = 0.46	Tau^2^ = 0.02

^***^p < 0.001, **p < 0.01, *p < 0.05

*Mixed-handedness:* The twin-to-singleton OR did not suggest a difference in mixed-handedness prevalence between singletons and twins (Table 1). There was evidence for heterogeneity among the studies (*p* < 0.05). Neither Egger’s regression test for funnel plot asymmetry (*z* = 0.90, *p* = 0.369), visual inspection of the funnel plot (Fig. 4b), nor the trim and fill test (0 studies to impute, SE = 1.43) revealed evidence for small study bias.

**Fig. 5:**
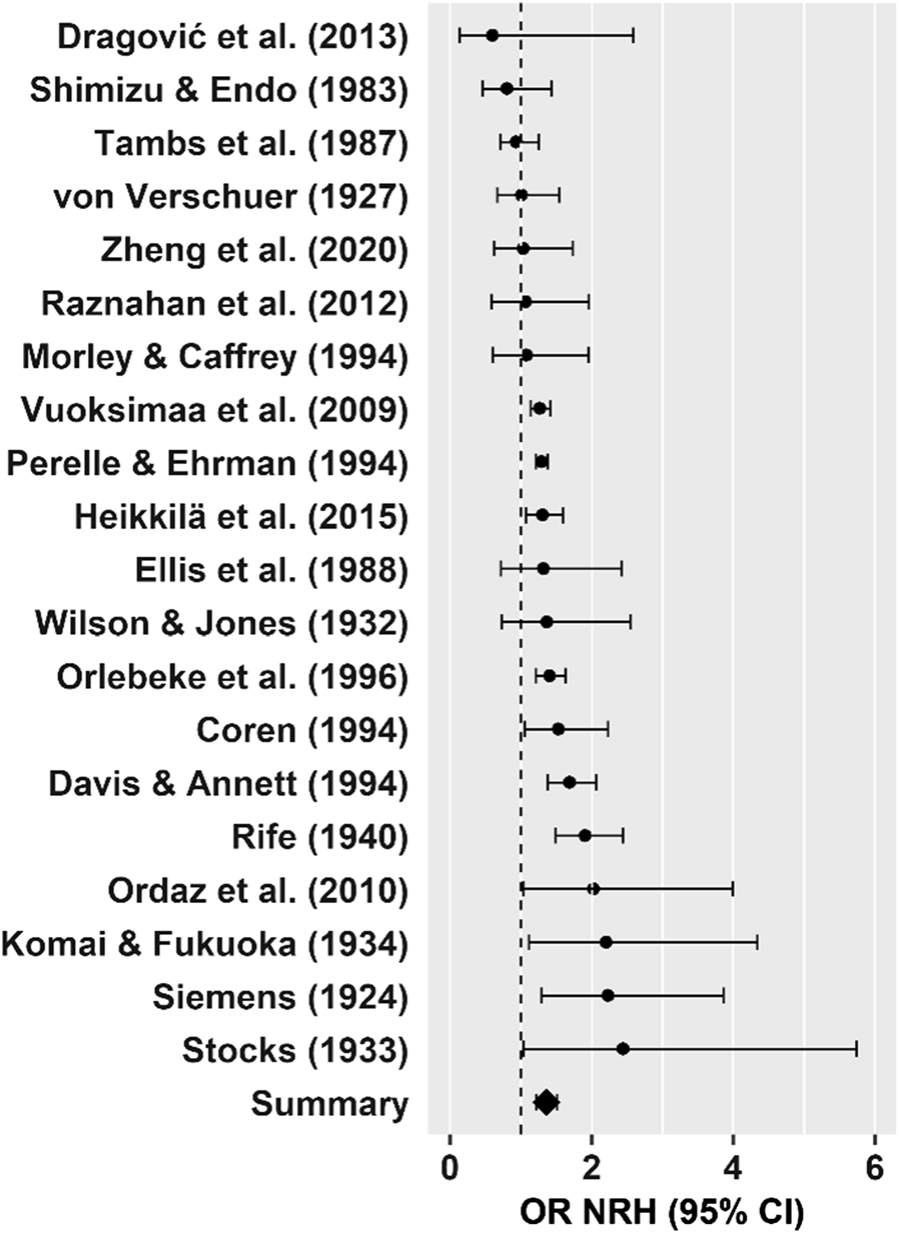
Forest plot for twin-to-singleton non-right-handedness meta-analysis. The dots represent ORs for each study and horizontal lines represent the 95% confidence intervals. The summary OR suggests higher prevalence of non-right-handedness in twins compared to singletons

*Non-right-handedness:* The twin-to-singleton OR suggested a higher prevalence of non-right-handedness in twins compared to singletons (Table 1, Fig. 5) with moderate to high heterogeneity among studies (*p* < 0.01). Neither Egger’s regression test (*z* = − 0.04, *p* = 0.967), nor visual inspection of the funnel plot (Fig. 4c) revealed evidence for small study bias. According to the trim and fill test, one study (SE = 2.85) needs to be imputed to the right of the mean for the funnel plot to be symmetrical. The resulting adjusted OR was 1.37 (95% CI = [1.23, 1.52], *z* = 5.74, *p* < 0.001).

*Moderator analyses:* Moderator analyses were conducted for both the non-right-handedness and the left-handedness classification scheme, but only the findings of the non-right-handedness classification are reported, as this was the most inclusive. We report results for the left-handedness classification in case the results differed between classification systems. In each moderator analysis, we included all studies for which the potential moderator variable could be extracted (see Table 2).

**Table 2:** Twin-to-singleton ORs in the different levels of the categorial moderator variables within the non-right-handedness (NRH) comparison. Overall, 20 studies were included in the NRH comparison (see main text).

Variable	Levels	Studies (*k*)	Participants (*n*)	Twins	Singletons	twin-to-singleton NRH OR [95% CI]
Sicotte et al. [10] meta-analysis	Yes (included in Sicotte et al. [10])	13	85,371	8281	77,090	1.43 [1.23, 1.66]
	No (new study)	6	38,394	30,773	7621	1.26 [1.01, 1.57]
Ancestry	Europe/USA/Australia	16	101,828	38,090	63,738	1.40 [1.23, 1.59]
	East Asia	3	21,937	964	20,973	1.16 [0.79, 1.72]
Study purpose	Handedness in twins	17	187,645	49,375	138,270	1.38 [1.23, 1.54]
	Other purpose	3	1629	691	938	1.21 [0.85, 1.74]
Type of singleton group	Genetically related to the twins	4	77,763	15,614	62,149	1.31 [1.07, 1.60]
	Genetically unrelated to the twins	12	103,122	33,799	69,323	1.41 [1.22, 1.63]
Handedness classification	R-A-L	4	38,975	26,511	12,464	1.12 [0.87, 1.45]
	R-L	12	83,177	11,852	71,325	1.45 [1.26, 1.68]
Method of handedness assessment	Preference obtained from inventories containing more than one item	5	16,721	1560	15,161	1.57 [1.26, 1.95]
	Self-reports/writing hand	12	170,689	47,700	122,989	1.32 [1.19, 1.47]

In cases where numbers do not add up to 20, some of the studies did not include information on the moderator variable

*Sicotte *et al*. (1999) meta-analysis:* First, we were interested if twin-to-singleton ORs differed between studies included in the meta-analysis by Sicotte et al. [10] (13 studies), studies specifically excluded from the meta-analysis by Sicotte et al. [10] (one study, which was thus excluded from this analysis), and new studies (six studies). There was no evidence for a difference in twin-to-singleton ORs between studies included in Sicotte et al. [10] and new studies, *Q*(1) = 0.86, *p* = 0.354.

**Fig. 6:**
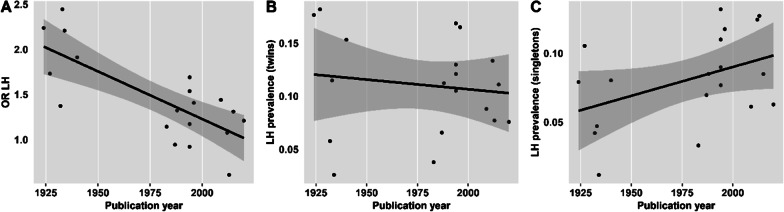
**a** Moderating effect of publication year on twin-to-singleton ORs for left-handedness. The twin-to-singleton OR for left-handedness decreases as the publication year of the individual study increases. This effect could be due to a decrease in left-handedness prevalence in twins, an increase in left-handedness prevalence in singletons, or both. **b** Association between publication year and left-handedness prevalence in twins. **c** Association between publication year and left-handedness prevalence in singletons

*Publication year:* There was no evidence for a moderating effect of publication year on twin-to-singleton ORs in the non-right-handedness classification, *Q*(1) = 3.52, *p* = 0.061. However, there was a significant effect of publication year on twin-to-singleton ORs in the left-handedness classification, *Q*(1) = 7.23, *p* < 0.01. The negative regression estimate (− 0.005, SE = 0.002, 95% CI = − 0.009, − 0.001) suggests smaller ORs in more recent studies (Fig. 6a).

To test whether both older and more recent studies show a higher prevalence of atypical handedness in twins compared to singletons, we ran separate random effects meta-analyses on studies published before 1975 (*k* = 6 studies including *n* = 21,372 singletons and *n* = 2290 twin individuals) and studies published after 1975 (*k* = 13 studies including *n* = 117,669 singletons and *n* = 47,591 twin individuals). The twin-to-singleton left-handedness OR was estimated to be 1.90 (95% CI = [1.59, 2.27], *z* = 6.98, *p* < 0.001) in studies published before 1975 and 1.30 (95% CI = [1.17, 1.45], *z* = 4.75, *p* < 0.001) in studies published after 1975.

Next, we were interested whether the decrease in ORs with publication year can be explained by an increase in left-handedness prevalence in singletons or a decrease of left-handedness prevalence in twins, or both. We ran random effects meta-analyses on the prevalence of left-handedness in twins and singletons separately and included publication year as a moderating variable. There was no evidence for a moderating effect of publication year on left-handedness prevalence in twins (*Q*(1) = 0.002, *p* = 0.968, Fig. 6b). There was, however, a trend towards higher left-handedness prevalence in more recent studies in singletons (*Q*(1) = 3.80, *p* = 0.051, Fig. 6c).

*Ancestry:* Next, we aimed to test for a moderating effect of ancestry. Ancestry was extracted from 19 studies reporting data on non-right-handedness and resulted in 16 studies of European/US American/Australian origin and three studies of East Asian origin. There was no evidence for a moderating effect of ancestry on twin-to-singleton ORs, *Q*(1) = 0.76, *p* = 0.383.

*Study purpose:* Next, we tested whether there was evidence for a moderating effect of whether the purpose of the original study was to examine the handedness in twins (17 studies) or not (three studies). There was no evidence for a moderating effect of purpose on twin-to-singleton ORs, *Q*(1) = 0.43, *p* = 0.510.

*Sex:* We tested whether sex ratio (extracted from nine studies) had any moderating effect. There was no evidence for a moderating effect of sex ratio on twin-to-singleton ORs, *Q*(1) = 0.20, *p* = 0.653.

*Mean age:* Likewise, there was no evidence for a moderating effect of mean age (extracted from seven studies) on twin-to-singleton ORs, *Q*(1) = 2.07, *p* = 0.151.

*Type of singleton group:* Furthermore, we investigated any potential effect of the type of singleton group on twin-to-singleton ORs. We distinguished between studies including singleton samples which were genetically related with the twin sample (four studies) and studies including singleton samples which were not genetically related with the twins (twelve studies). There was no evidence for a moderating effect of singleton group type on twin-to-singleton ORs, *Q*(1) = 0.37, *p* = 0.541.

*Handedness classification:* Next, we investigated a potential moderating effect of handedness classification, divided into “R-A-L” (four studies) and “R-L” (twelve studies). There was no evidence for a moderating effect of classification on twin-to-singleton ORs, *Q*(1) = 3.03, *p* = 0.082.

*Method of handedness assessment:* Last, we aimed to reveal potential moderating effects of the method of handedness assessment. To this end, we compared preference obtained from performance inventories in which the individuals’ handedness was determined on the basis of more than one item (five studies) and self-reports/writing hand (twelve studies). There was no evidence for a moderating effect of handedness assessment on twin-to-singleton ORs, *Q*(1) = 1.87, *p* = 0.171.

### Meta-analysis set 2: prevalence of atypical handedness in MZ vs. DZ

In our second set of meta-analyses, we aimed to investigate whether there was a difference in the prevalence of atypical handedness between DZ and MZ twins. Overall, 48 studies allowed for the calculation of MZ-to-DZ ORs, including *n* = 36,043 DZ individuals and *n* = 27,252 MZ individuals, resulting in a total sample size of *n* = 63,295 individuals.

*Left-handedness:* The MZ-to-DZ OR revealed no evidence for a difference in left-handedness prevalence between MZ and DZ twins (Table 3). Heterogeneity among the studies was moderate (*p* = 0.002). Neither Egger’s regression test for funnel plot asymmetry (*z* = 1.34, *p* = 0.182), nor visual inspection of the funnel plot (Fig. 4d) revealed evidence for small study bias. However, according to the trim and fill test, six studies (SE = 4.32) would need to be imputed to the left of the mean in order for the funnel plot to be symmetrical. The resulting adjusted OR was 0.94 (95% CI = [0.85, 1.03], *z* = − 1.35, *p* = 0.178).

**Table 3:** Results of meta-analysis set 2

	Left-handedness	Mixed-handedness	Non-right-handedness
Studies (*k*)	43	10	47
	27 included in [10]	5 included in [10]	28 included in [10]
	3 excluded from [10]	5 new studies	3 excluded from [10]
	13 new studies		16 new studies
Individuals in total (*n*)	63,295		
Individuals per comparison (*n*)	59,973	28,511	63,181
MZ twins (*n*)	25,957	10,164	27,203
DZ twins (*n*)	34,016	18,347	35,978
Prevalence in MZ twins	11.45% (*n* = 2971)	1.83% (*n* = 186)	12.08% (*n* = 3286)
Prevalence in DZ twins	11.82% (*n* = 4019)	3.26% (*n* = 599)	13.29% (*n* = 4780)
OR [95% CI]	0.98 [0.89, 1.07]	0.96 [0.46, 1.99]	1.01 [0.91, 1.12]
*z*	− 0.51	− 0.11	0.13
Heterogeneity among studies	Q(42) = 74.08**	Q(9) = 100.52***	Q(46) = 149.78***
	I^2^ = 36.00%	I^2^ = 86.33%	I^2^ = 57.63%
	Tau^2^ = 0.02	Tau^2^ = 0.88	Tau^2^ = 0.05

***p < 0.001, **p < 0.01

*Mixed-handedness:* The MZ-to-DZ mixed-handedness OR did not provide evidence for a difference in mixed-handedness prevalence between MZ and DZ twins (Table 3). Heterogeneity among the studies was high (*p* < 0.001). Neither Egger’s regression test for funnel plot asymmetry (*z* = 1.49, *p* = 0.137), nor visual inspection of the funnel plot (Fig. 4e) revealed evidence for small study bias. According to the trim and fill test, four studies (SE = 2.02) would need to be imputed to the left of the mean in order for the funnel plot to be symmetrical. The resulting adjusted OR was 0.61 (95% CI = [0.31, 1.20], *z* = − 1.43, *p* = 0.152).

*Non-right-handedness:* The MZ-to-DZ non-right-handedness OR did not provide evidence for a difference in non-right-handedness prevalence between MZ and DZ twins (Table 3). Heterogeneity among the studies was moderate (*p* < 0.001). Neither Egger’s regression test for funnel plot asymmetry (*z* = 1.73, *p* = 0.083), nor visual inspection of the funnel plot (Fig. 4f) revealed evidence for small study bias. However, according to the trim and fill test, eight studies (SE = 4.54) would need to be imputed to the left of the mean in order for the funnel plot to be symmetrical. The resulting adjusted OR was 0.94 (95% CI = [0.84, 1.06], *z* = − 1.00, *p* = 0.320).

*Moderator analysis:* There was no evidence for a difference in MZ-to-DZ non-right-handedness ORs between studies included in Sicotte et al. [10] (28 studies), studies excluded by Sicotte et al. [10] (three studies) and new studies (16 studies), *Q*(2) = 0.75, *p* = 0.687.

We then investigated a potential moderating effect of the method used to determine zygosity on MZ-to-DZ ORs. Studies were divided into “serological and genetic analyses” (eleven studies) and “questionnaire” (25 studies). There was no evidence for a moderating effect of the method used to determine zygosity on MZ-to-DZ non-right-handedness ORs, *Q*(1) = 0.06, *p* = 0.809.

### Meta-analysis set 3: concordance of handedness in MZ vs. DZ

The aim of our third set of meta-analyses was to test whether DZ and MZ twin pairs differed in pairwise handedness concordance. Overall, 44 studies (27 included by Sicotte et al. [10], one study excluded by Sicotte et al. [10], 16 new studies) allowed for the calculation of ORs for pairwise concordance in MZ vs. DZ twins, including *n* = 20,711 DZ twin pairs and *n* = 15,506 MZ twin pairs, resulting in a total sample size of *n* = 36,217 twin pairs. Across all studies, the concordance rate was 80.49% in MZ twin pairs (*n* = 12,481 concordant twin pairs) and 79.27% in DZ twin pairs (*n* = 16,417 concordant twin pairs).

**Fig. 7:**
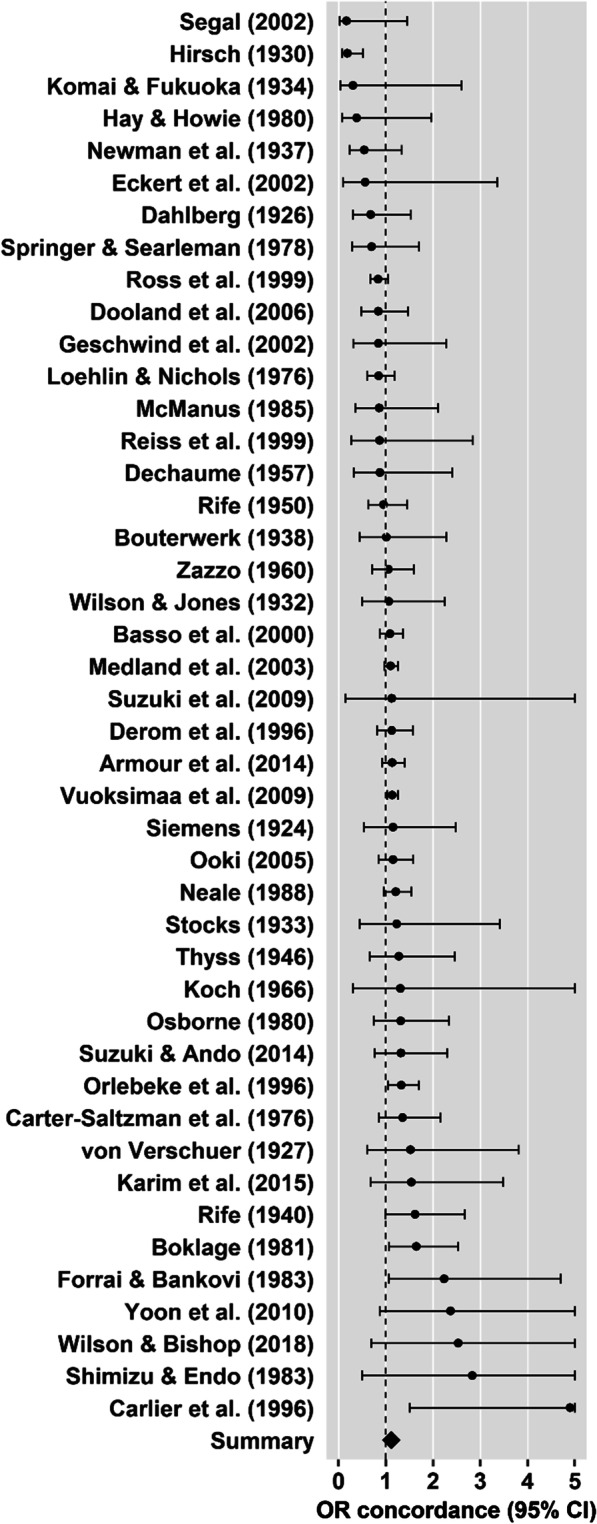
Forest plot for MZ-to-DZ concordance meta-analysis. The dots represent ORs for each study and horizontal lines represent the 95% confidence intervals. The summary OR suggests a slightly higher handedness concordance in MZ twins compared to DZ twins

The concordance OR was estimated to be 1.11 (95% CI = [1.06, 1.18], *z* = 3.91, *p* < 0.001, Fig. 7). Heterogeneity among the studies was low, *Q*(43) = 60.01, *p* < 0.05, *I*^*2*^ = 0.02%, Tau^2^ = 0.00. Neither Egger’s regression test for funnel plot asymmetry (*z* = − 0.54, *p* = 0.590), nor visual inspection of the funnel plot (Fig. 4g) revealed evidence for small study bias. According to the trim and fill test, one study (SE = 4.10) would need to be imputed to the right of the mean in order for the funnel plot to be symmetrical. The resulting adjusted OR was 1.12 (95% CI = [1.06, 1.18], *z* = 3.96, *p* < 0.001).

There was no evidence for a difference in MZ-to-DZ concordance ORs between studies included by Sicotte et al. [10] (27 studies), excluded by Sicotte et al. [10] (one study, which was thus excluded from this analysis), and new studies (16 studies), *Q*(1) = 0.88, *p* = 0.349.

Likewise, there was no evidence for a moderating effect of the method used to determine zygosity on concordance ORs, *Q*(1) = 0.04, *p* = 0.834, suggesting that there was no difference between studies using genetic and/or serological analyses (twelve studies) and studies using questionnaire methods (22 studies) to determine zygosity.

## Discussion

In three sets of meta-analyses, we examined the influence of twin status and twin zygosity on handedness prevalence and handedness concordance. Our first set of meta-analyses confirmed that in line with Sicotte et al. [10], left-handedness (OR = 1.40, Fig. 3) and non-right-handedness (OR = 1.36, Fig. 5) occur more often among twins than among singletons. Moderator analyses found elevated levels of non-right-handedness among twins to be independent of all variables tested with respect to a potential moderating effect. However, we found that more recent studies reported smaller differences in prevalence of left-handedness between twins and singletons (Fig. 6). To test whether there is a higher left-handedness prevalence in twins compared to singletons in more recent studies at all, we estimated twin-to-singleton ORs for left-handedness for studies published pre and post 1975 separately. With a pre-1975 OR of 1.90 (95% CI = [1.59, 2.27]) and a post-1975 OR of 1.30 (95% CI = [1.17, 1.45]), ORs for more recent studies were smaller, but still indicated a significant twin effect on left-handedness.

Overall, the decrease in twin-to-singleton ORs might either be explained by a decrease in left-handedness in twins or an increase of left-handedness in singletons, or both. As already mentioned, complications occur more often in the course of multiple births [11–13], which might contribute to the development of atypical handedness [10]. However, most individual studies included in our meta-analysis did not provide information on pre- or perinatal conditions, so we could not test for a moderating effect of these conditions on the twin-to-singleton OR. Along these lines, future research might have a closer look on the relation between birth complications and handedness.

Assuming that higher proportions of left-handedness among twins might be the by-product of birth complications, a decrease in atypical handedness in twins must be assigned to a decrease in the occurrence of these complications. In fact, it is well conceivable that medical progress over the last decades, that is clearly detectable, e.g. in the United States [92, 93], may have helped to equalize the risks associated with multiple and single births. Such assumptions are supported by a study by Heikkilä et al. [77] who showed differences in left-handedness in twins and singletons to disappear when controlling for birth weight, Apgar score, and gestational age. We therefore tested whether there is evidence for a decrease in left-handedness prevalence in twins (Fig. 6b) by running meta-analyses on left-handedness prevalence in twins and singletons separately while including publication year as a moderator variable. However, while there was no evidence for an effect of publication year on left-handedness prevalence in twins, there seemed to be a trend towards an increase of left-handedness prevalence in singletons (Fig. 6c).

The overall prevalence of atypical handedness in our study was lower than expected. We found 9.13% of twins and 6.97% of singletons to be left-handed (Table 1), while Papadatou-Pastou et al. [3] reported a figure of 10.6% (95% CI 9.71%, 11.50%) for the general population. The low values in our study might be the result of a general effect of publication year in singletons, given that the prevalence of left-handedness has been shown to be higher in younger than in older cohorts [25, 94, 95]. The social stigma associated with left-handedness in the last century [96] may have driven left-handers to conceal their preference in self-reports [97] and to retrain to use their right hand [25, 98]. Most of the studies included in our meta-analysis were published in the previous century and their participants could have been subjected to environmental pressures against left-handedness, leading to underestimation of the true population prevalence of left-handedness. Similarly, we found low overall prevalence of mixed-handedness (3.39% in twins and 2.67% in singletons, Table 1), whereas Papadatou-Pastou et al. [3] gave a point estimate of 9.3% for the general population. This might also be due to an effect of publication year. Moreover, three of five studies that provided data for mixed-handedness classified handedness as writing hand so that data extracted from these studies most likely reflect not mixed-handedness, but ambidexterity, which is much rarer [99].

Our second set of meta-analyses found no difference in the prevalence of atypical handedness between MZ and DZ twins (left-handedness OR = 0.98, mixed-handedness OR = 0.96, non-right-handedness OR = 1.01, Table 3). This result is consistent with the meta-analysis by Sicotte et al. [10] who interpreted this null-effect as indication against mirror imaging theories designed to explain heightened frequencies of left-handers and frequent handedness discordance among MZ twins [100–102]. Indeed, it weakens the hypothesis suggesting that the monozygotic twinning process is responsible for atypical handedness [10]. Moreover, it indicates that the overall heightened frequencies of left- and non-right-handers among twins are independent of the twins’ zygosity. A moderator analysis showed that this effect was not influenced by the method used to determine the twins’ zygosity, thus refuting the idea that the result was affected by the accuracy with which twins were classified as monozygotic or dizygotic. All in all, revealing comparable prevalence of atypical handedness for MZ and DZ twins cannot enrichen knowledge about genetic contribution to handedness per se. As already recognized by Sicotte et al. [10], to do so, it is crucial to look at pairwise handedness concordance or discordance of MZ and DZ twin pairs.

Our third set of meta-analyses found a small yet significant effect (OR = 1.11, Fig. 7) for higher handedness concordance among MZ (80.49%) as compared to DZ (79.27%) twins, consistent with the meta-analysis by Sicotte et al. [10]. Even though other publications have demonstrated the occurrence of handedness discordance among MZ twin pairs [100, 101, 103, 104], it was estimated to concern a minority of 20–25% of cases [2]. Stronger phenotypic variation among DZ compared to MZ pairs indicates a certain genetic foundation of that phenotype [2, 26]. Therefore, our results confirm handedness to rely on genetic factors to some extent [10] and are consistent with heritability estimates of 0.24–0.26 [31–33]. A moderator analysis suggested that the frequencies of handedness concordance did not differ between studies included in the meta-analysis by Sicotte et al. [10], studies explicitly excluded from Sicotte et al. [10], and more recent studies.

To allow future meta-analyses to perform comparisons on handedness prevalence in twins more specifically (e.g., handedness in male vs. female twins, or handedness in same sex pairs vs. opposite sex pairs), it is desirable that researchers report results broken down for parameters like zygosity, sex, and consider data on birth complications. As this might be beyond the scope of individual papers, we encourage authors to provide open raw data in publicly accessible repositories such as the osf.io.

The present study is not without limitations. We did not investigate relative hand skill but were restricted to hand preference. Measuring hand preference is far more established as compared to assessing relative hand skill, as it is easier and more convenient [105]. Most of the studies included in our meta-analysis only provided information on hand preference, not allowing for an additional analysis for hand skill. Moreover, hand preference and hand skill correlate to some extent [106–108], and the distribution of handedness categories overlaps for preference- and skill-related criterions in 90% of the cases [109].

Similarly, our study only dealt with handedness direction in terms of categorial handedness classification which does not take into account the fact that individual handedness can further be defined regarding its strength or its degree. Along these lines, other approaches consider handedness as a continuum, extending the question to how strong or how consistently one hand is preferred, used, or skilled over the other. Indeed, several findings obtained within laterality research on associations between handedness and structural brain lateralization [110] or cognitive performance [111, 112] as well as concerning the genetic foundation of handedness [113, 114] are linked to strength but not direction of handedness. However, since most studies included in the present meta-analyses did not assess handedness in a continuous manner, we were unable to account for handedness strength. Therefore, it falls to future studies to extend their assessment repertoire by measures of handedness strength.

From a methodological point of view, it is further crucial to mention that overall, our moderator analyses are low in power due to the investigated study sample sizes. Of note, in some cases, moderator levels included only three data points calling for an interpretation of these findings with caution.

## Conclusion

To summarize, our analyses provide evidence for increased frequencies of left- and non-right-handedness among twins compared to singletons but do not support the notion of elevated prevalence of atypical handedness among MZ compared to DZ twins. Therefore, our findings are in line with the interpretation that twin or multiple births may be accompanied by certain environmental conditions that disturb the establishment of right-handedness. Moreover, our analysis showed that the prevalence of atypical handedness seems to be steadily equalizing for twins and singletons over time. Indeed, the last decades may have advanced medical progress so that the occurrence of risks associated twin births that mediate the shift towards non-right-handedness is aligned with the occurrence of these risks within single births. However, separate analysis in twins and singletons suggests that this effect is rather the product of an increase of left-handedness prevalence in singletons rather than a decrease of left-handedness prevalence in twins. As we further showed MZ twins to be more frequently handedness concordant than DZ twins, we can confirm a partially genetic foundation of phenotypic handedness which, however, does not seem to account for the vast majority of this trait. We generally acknowledge phenotypic handedness to arise from a complex interaction of genetic and environmental influences that can only be understood by means of multi-level approaches. Specifying how handedness evolves should finally serve to comprehend the population level predominance of right-handedness as well as the overrepresentation of atypical handedness in samples like twins.

## Supplementary Information

**Additional file 1.** Supplementary table 1.

**Additional file 1.** PRISMA guidelines.

## Data Availability

All data and analysis codes are available in the OSF project “Handedness in Twins: Meta-Analyses” under the link: https://osf.io/w7jem/ (the project was preregistered under the link: https://osf.io/ywhsj). Analyses were conducted as planed in the preregistration and there were no deviations from the preregistered research protocol.

## Abbreviations

DZ: Dizygotic
EHI: Edinburgh handedness inventory
MZ: Monozygotic
OR: Odds ratio
LH: Left-handedness
MH: Mixed-handedness
NRH: Non-right-handedness
