# Supplementary material for: Handedness in twins: meta-analyses
Source: BMC Psychol. 2022 Jan 15;10:11. doi: 10.1186/s40359-021-00695-3 (PMC8760823; doi:10.1186/s40359-021-00695-3)
Supplement: Supplementary file 1 — Additional file 1. Supplementary table 1. [file 40359_2021_695_MOESM1_ESM.pdf]

Supplementary Material: Handedness in Twins: Meta-Analyses

Lena Sophie Pfeifer, Judith Schmitz, Marietta Papadatou-Pastou, Jutta Peterburs, Silvia Paracchini, Sebastian Ocklenburg

Table S1. Comprehensive list of all studies included in our meta-analyses. It is further reported in which of our three sets of meta-analyses the study was included and where we found it.

Citation	Title of Study	Set of meta-analyses	Sicotte et al. (1999) meta-analysis / Source
Boklage [115]	On the distribution of non-right-handedness among twins and their families	set 2, set 3	Sicotte (included)
Carlier et al. [91]	Manual performance and laterality in twins of known chorion type	set 2, set 3	Sicotte (included)
Carter-Saltzman et al. [116]	Left-handedness in twins: Incidence and patterns of performance in an adolescent sample	set 2, set 3	Sicotte (included)
Coren [117]	Twinning is associated with an increased risk of left-handedness and inverted writing hand posture	set 1	Sicotte (included)
Dahlberg [118]	Twin births and twins from a hereditary point of view	set 2, set 3	Sicotte (included)
Davis et al. [5]	Handedness as a function of twinning, age and sex	set 1	Sicotte (included)
Dechaume [83]	Contribution a l'étude de la dominance laterale chez les jumeaux	set 2, set 3	Sicotte (included)

Derom et al. [119]	Handedness in twins according to zygosity and chorion type: A preliminary report	set 2,<br>set 3	Sicotte (included)
Ellis et al. [9]	Hand preference in a normal population	set 1	Sicotte (included)
Forrai et al. [120]	A Hungarian twin study on hand clasping, arm folding and tongue curling	set 2,<br>set 3	Sicotte (included)
Hay et al. [121]	Handedness and differences in birthweight of twins	set 2,<br>set 3	Sicotte (included)
Hirsch [85]	Twins, heredity and environment	set 2,<br>set 3	Sicotte (included)
Koch [86]	Twins and twin relations	set 2,<br>set 3	Sicotte (included)
Komai et al. [102]	A note on the problem of mirror-imaging in human twins	set 1,<br>set 2,<br>set 3	Sicotte (included)
Loehlin et al. [122]	Heredity, environment and personality: A study of 850 sets of twins	set 2,<br>set 3	Sicotte (included)
McManus [123]	Handedness, language dominance and aphasia: A genetic model	set 2,<br>set 3	Sicotte (included)
Morley et al. [124]	Handedness in blood donors: No association with blood group or twinning	set 1	Sicotte (included)
Neale [125]	Handedness in a sample of volunteer twins	set 2,<br>set 3	Sicotte (included)
Newman et al. [126]	Twins, a study of heredity and environment	set 2,	Sicotte (included)

		set 3	
Orlebeke et al. [127]	Left-handedness in twins: Genes or environment?	set 1, set 2, set 3	Sicotte (included)
Osborne [128]	Twins: Black and white	set 2, set 3	Sicotte (included)
Rife [6]	Handedness, with special reference to twins	set 1, set 2, set 3	Sicotte (included)
Rife [129]	An application of gene frequency analysis to the interpretation of data from twins	set 2, set 3	Sicotte (included)
Shimizu et al. [88]	Comparison of patterns of handedness between twins and singletons in Japan	set 1, set 2, set 3	Sicotte (included)
Siemens [130]	Über Linkshändigkeit	set 1, set 2, set 3	Sicotte (included)
Springer et al. [131]	Laterality in twins: The relationship between handedness and hemispheric asymmetry for speech	set 2, set 3	Sicotte (included)
Stocks [101]	A biometric investigation of twins and their brothers and sisters	set 1, set 2, set 3	Sicotte (included)
Tambs et al. [8]	Left-handedness in twin families: Support of an environmental hypothesis	set 1, set 2	Sicotte (included)

Thyss [84]	Etude bibliographique et critique du problème des gauchers	set 2,<br>set 3	Sicotte (included)
von Verschuer [132]	Die vererbungsbiologische Zwillingsforschung	set 1,<br>set 2,<br>set 3	Sicotte (included)
Wilson et al. [133]	Left-handedness in twins	set 1,<br>set 2,<br>set 3	Sicotte (included)
Zazzo [28]	Les jumeaux: Le couple et la personne	set 2,<br>set 3	Sicotte (included)
Bouterwerk [59]	Rechts-Links-Abwandlung in Händigkeit and seelischer Artung	set 2,<br>set 3	Sicotte (excluded)
Liebing [62]	Zur Verteilung der Händigkeit bei Vorschulkindern	set 2,<br>set 3	Sicotte (excluded)
Perelle et al. [60]	An international study of human handedness: The data	set 1	Sicotte (excluded)
Shields [61]	Monozygotic twins: Brought up apart and brought up together	set 2,<br>set 3	Sicotte (excluded)
Armour et al. [134]	Genome-wide association study of handedness excludes simple genetic models	set 3	new study
Basso et al. [82]	Handedness and mortality: a follow-up study of Danish twins born between 1900 and 1910	set 2,<br>set 3	new study
Bohlken et al. [135]	Heritability of structural brain network topology: a DTI study of 156 twins	set 2	new study

Dooland et al. [81]	Prevalence and side preference for tooth grinding in twins	set 2, set 3	new study
Dragović et al. [136]	Etiological aspect of left-handedness in adolescents	set 1	new study
Eckert et al. [137]	The epigenesis of planum temporale asymmetry in twins	set 2, set 3	new study
Geschwind et al. [138]	Heritability of lobar brain volumes in twins supports genetic models of cerebral laterality and handedness	set 2, set 3	new study
Heikkilä et al. [77]	Higher prevalence of left-handedness in twins? Not after controlling birth time confounders	set 1	new study
Hulshoff Pol et al. [67]	Genetic contributions to human brain morphology and intelligence	set 1, set 2	new study
Karim et al. [139]	Human lateralization among monozygotic and dizygotic twins in Koya Town	set 2, set 3	new study
Medland et al. [36]	Special twin environments, genetic influences and their effects on the handedness of twins and their siblings	set 2, set 3	new study
Ooki [140]	Genetic and environmental influences on the handedness and footedness in Japanese twin children	set 3	new study
Ordaz et al. [141]	Are there differences in brain morphometry between twins and unrelated singletons? A pediatric MRI study	set 1	new study

Raznahan et al. [142]	Reply to Segal: Are relationships between birth weight and intelligence quotient variation within twin pairs modulated by patterns of handedness discordance?	set 1, set 2	new study
Reiss et al. [143]	Laterality of hand, foot, eye, and ear in twins	set 2, set 3	new study
Ross et al. [144]	Handedness in the NAS/NRC twin study	set 2, set 3	new study
Segal [145]	Co-conspirators and double-dealers: a twin film analysis	set 2, set 3	new study
Suzuki et al. [146]	Genetic and environmental structure of individual differences in hand, foot, and ear preferences: A twin study	set 2, set 3	new study
Suzuki et al. [147]	Genetic effects on infant handedness under spatial constraint conditions	set 2, set 3	new study
Vuoksimaa et al. [69]	Origins of handedness: a nationwide study of 30,161 adults	set 1, set 2, set 3	new study
Wilson et al. [56]	Resounding failure to replicate links between developmental language disorder and cerebral lateralisation	set 2, set 3	new study
Yoon et al. [148]	Lateralized genetic and environmental influences on human brain morphology of 8-year-old twins	set 2, set 3	new study
Zheng et al. [35]	Prevalence and heritability of handedness in a Hong Kong	set 1	new study

	Chinese twin and singleton sample		
